# A Phenotypically Normal Homozygous Balanced Reciprocal Translocation Carrier: Report of an Extremely Rare Genetic Occurrence

**DOI:** 10.7759/cureus.93614

**Published:** 2025-09-30

**Authors:** Mitila Thirupathy, Mitesh Shetty, Priya Prakash

**Affiliations:** 1 Medical Genetics, Manipal Hospital, Bengaluru, IND; 2 Medical Genetics, Manipal College of Health Professions, Manipal Academy of Higher Education (MAHE), Bengaluru, IND

**Keywords:** balanced reciprocal translocation, homozygous, infertility, pgt-sr, segregation patterns

## Abstract

Balanced reciprocal translocations (BRT) are relatively common structural chromosomal abnormalities and are typically observed in the heterozygous state. Homozygosity for reciprocal translocations is exceedingly rare, with most documented cases presenting with severe congenital anomalies or developmental delays. To the best of our knowledge, this is the second reported case of a phenotypically normal homozygous BRT carrier born from a natural conception. A 31-year-old female with primary infertility was found to be homozygous for the balanced reciprocal translocation t(11;22)(q23.3;q11.2) during assessment after a failed cycle of in vitro fertilization. Although homozygosity for such translocations can theoretically lead to meiotic errors, gene disruption, or recessive disorders, the proband exhibited a normal phenotype, likely owing to the completely balanced nature of the translocation, making this an exceptionally rare occurrence, challenging assumptions about the consequences of homozygous translocations, and highlighting the need for further research into the specific breakpoint regions.

## Introduction

Balanced reciprocal translocations (BRT) involve exchanging chromosomal segments between non-homologous chromosomes without genetic material loss or gain, occurring in ~0.2% of the population [1]. Heterozygous BRT carriers are usually phenotypically normal but have higher risks of infertility, pregnancy loss, or offspring with unbalanced rearrangements due to meiotic malsegregation [2,3]. The t(11;22)(q23;q11.2) translocation is the most commonly documented recurrent non-Robertsonian rearrangement [4]. Individuals with homozygous translocation, inheriting identical translocations from both parents, are rare, often showing severe congenital anomalies or developmental delays due to gene disruption or cryptic imbalances [5-7]. Consanguinity increases this likelihood by raising the probability of both parents carrying the same translocation [8-10]. We describe a 31-year-old phenotypically normal female who was found to be homozygous for t(11;22)(q23.3;q11.2), an extraordinarily rare phenomenon. This unusual finding emphasizes the potential for completely balanced nature of chromosomal rearrangement in situations where reciprocal translocations are present in a homozygous state. It also reinforces the importance of genetic counseling for couples at risk, particularly in guiding reproductive options and assessing potential outcomes.

## Case presentation

**Figure 1:**
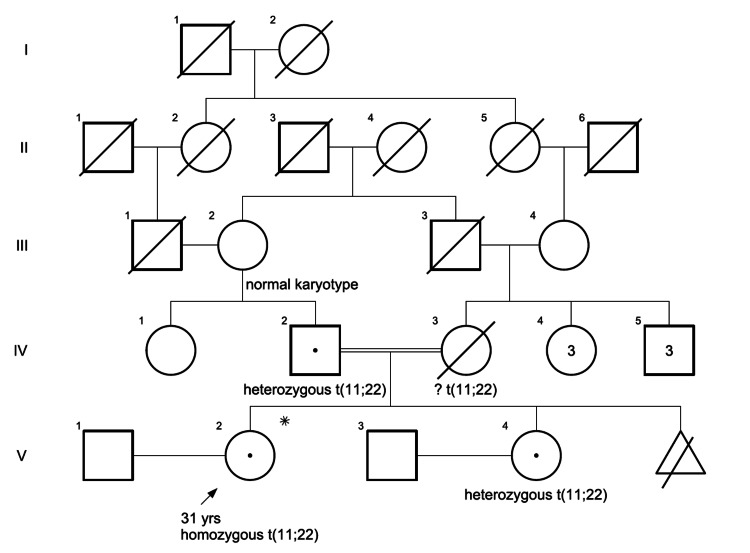
Pedigree of our t(11;22)(q23;q11.2) family. Proband (V-2), born of consanguineous parentage, is a homozygous carrier of the translocation. The sibling (V-4) and father (IV-2) are heterozygous carriers of the same translocation. Paternal grandmother (III-3; shown as unfilled circle), the sibling of maternal grandfather (III-c), showed a normal karyotype. Carriers are represented with a dot in the circle/square.

A 31-year-old female presented with primary infertility. She had no relevant past medical history, including congenital, developmental, or other health conditions. Family history revealed consanguineous parentage (first cousins), a maternal history of premenopausal breast cancer (BC) affecting her mother and mother’s one of three sisters in their early thirties, and complex familial consanguinity (Figure 1). The proband’s mother, deceased at 47 years of age, had a history of medical termination of the third conceptus due to a congenital abnormality.

**Figure 2:**
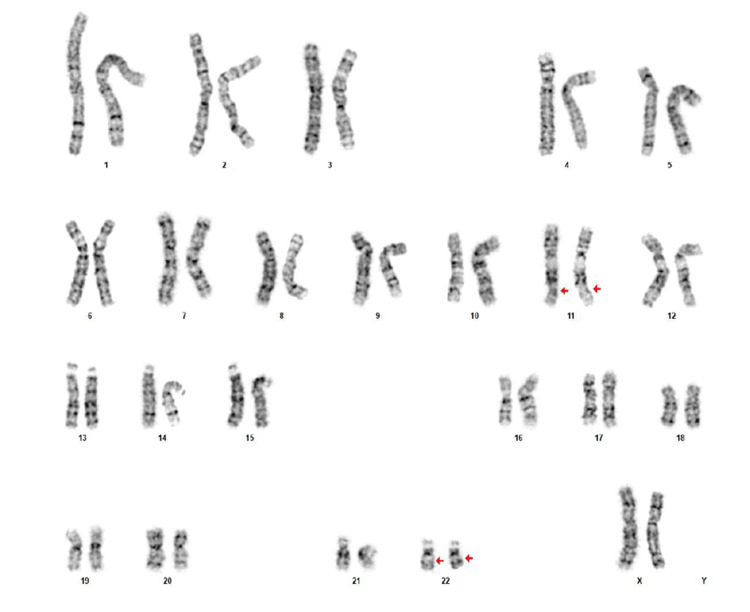
Karyogram of the proband with a band resolution 550, revealing the karyotype of 46,XX,t(11;22)(q23.3;11.2),t(11;22)(q23.3;11.2).

She had undergone failed intrauterine insemination (IUI) followed by a cycle of in vitro fertilization (IVF). IVF yielded only two embryos that were subjected to preimplantation genetic testing for aneuploidies (PGT-A) analysis by next-generation sequencing, both of which were aneuploid and not recommended for transfer. Couple karyotyping was advised, and the patient’s karyotype revealed a homozygous balanced reciprocal translocation (Figure 2).

**Figure 3:**
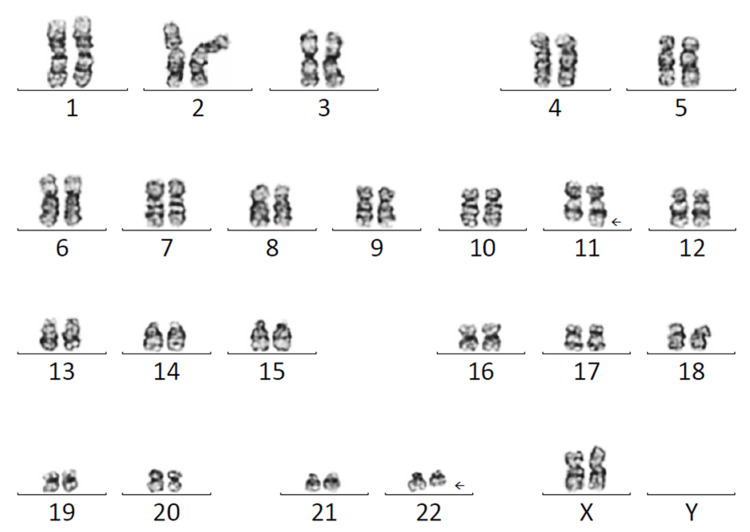
Karyogram of the proband’s sibling, with a band resolution of 450, revealing karyotype of 46,XX,t(11;22)(q23.3;11.2).

Chromosomal microarray (CMA) testing by whole genome single-nucleotide polymorphism (SNP) microarray analysis was normal, with no significant copy number changes at the translocation breakpoints. Carrier screening for 282 diseases was negative. Upon subsequent testing, the patient’s father and sibling were heterozygous carriers of the same translocation (Figure 3), while the paternal grandmother had a normal karyotype.

Preimplantation genetic testing for structural rearrangements (PGT-SR) with IVF was recommended to select balanced embryos, supplemented by CMA, and uniparental disomy (UPD) testing at 16-20 weeks of gestation.

## Discussion

**Figure 4:**
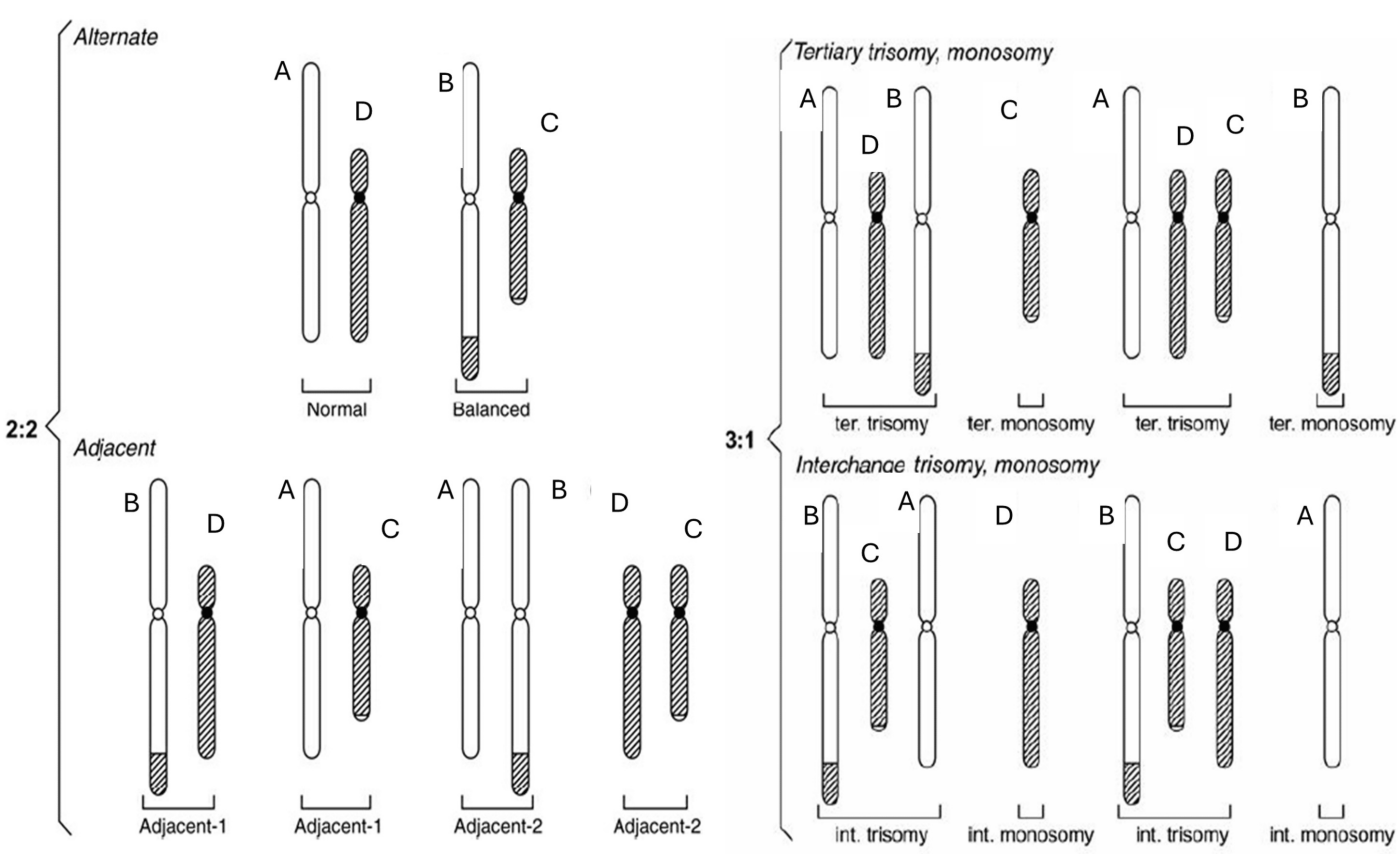
Segregation patterns of a translocation heterozygote Image courtesy: Gardner RJM and Amor DJ [11]

Homozygous reciprocal translocations are among the rarest genetic phenomena, with only a few documented cases in the medical literature [5-7, 10]. The translocation breakpoints were mapped to AT-rich inverted repeat regions, with palindromic sequences on chromosomes 11 and 22 suggested to play a crucial role in its formation [1,4]. Heterozygous carriers of t(11;22) are generally phenotypically normal but face a 5-10% risk of producing chromosomally unbalanced offspring due to 2:2 or 3:1 meiotic malsegregation, often resulting in conditions such as Emanuel syndrome [2,3]. Theoretically, a heterozygous carrier of an autosomal BRT can produce 16 distinct chromosomal combinations in their gametes, however, 4:0 segregants are typically non-viable* (Figure 4, Table 1) [11]. Consequently, the likelihood of two heterozygous parents, each carrying the same translocation, producing a homozygous offspring is one in 196, derived from the 14 viable gamete combinations per parent [11,12]. Among the 196 possible outcomes, 15 of them result in balanced chromosomal products, of which 12 are associated with UPD, while the remaining three include two balanced heterozygous outcomes and one balanced homozygous translocation [9].

**Table 1:** Patterns of segregation of a reciprocal translocation Courtesy: Turnpenny PD, Ellard S, Cleaver R [12]

Pattern of Segregation	Segregating Chromosomes	Chromosome Constitution in Gamete
2:2		
Alternate	A + D	Normal
	B + C	Balanced translocation
Adjacent-1 (non-homologous centromeres segregate together)	A + C or B + D	Unbalanced, leading to partial monosomy and partial trisomy in the zygote
Adjacent-2 (homologous centromeres segregate together)	A + B or C + D	
3:1		
Three chromosomes	A + B + C	Unbalanced, leading to trisomy in the zygote
	A + B + D	
	A + C + D	
	B + C + D	
One chromosome	A	Unbalanced, leading to monosomy in the zygote
	B	
	C	
	D	
4:1 *		
Four chromosomes	A + B + C + D	Unbalanced, leading to double trisomy in the zygote
Zero Chromosomes	-	Unbalanced, leading to double monosomy in the zygote

Most documented cases of homozygous translocations have been characterized by significant phenotypic abnormalities. For instance, Martinet et al. described a 24-week fetus with multiple organ anomalies, including Pierre-Robin sequence, cardiac defects, and central nervous system heterotopias, homozygous for a t(17;20) translocation [5]. The authors suggested that gene disruption at breakpoints or recessive mutations unmasked by consanguinity contributed to the severe phenotype. Similarly, Wilmot et al. reported an infant with a disomic t(3;16) translocation who presented with infantile seizures, hypotonia, and minor dysmorphic features, potentially due to homozygous gene loss or consanguinity-related homozygosity [6]. To the best of our knowledge, Koç et al. reported the first case of a healthy homozygous balanced translocation carrier from natural conception, a t(5;16) carrier born to first-cousin parents, with recurrent fetal losses, whose heterozygous offspring showed no abnormalities [7]. Our case constitutes only the second such reported instance with a normal phenotype, emphasizing its uncommon nature. This variability in phenotypic outcomes highlights the complex interplay of genetic factors, including the specific chromosomes involved, breakpoint locations, consanguinity, and other influences.

Consanguinity significantly raised the risk of a shared translocation. Simoni et al. documented a similar scenario, where first-cousin parents with identical t(2;7) translocations produced phenotypically normal offsprings, all of whom inherited the translocation (heterozygous carriers) [8]. Cryptic deletions, detected in 40% of phenotypically abnormal translocation cases, were absent in our proband, as confirmed by CMA [13]. Tapia-Páez et al. noted that t(11;22) breakpoints at palindromic repeats rarely disrupt genes, possibly explaining the lack of phenotypic effects in some carriers [4].

The proband’s primary infertility is likely due to meiotic instability, a recognized consequence of BRT [2,3]. Although homozygous carriers may predominantly produce aneuploid gametes due to factors such as altered imprinting, interchromosomal effects, or gene disruption at the translocation breakpoints [6,7,10], the euploid gametes capable of resulting in successful live births are expected to be fully balanced. Beyazyurek et al. demonstrated the efficacy of PGT-SR in a consanguineous couple with identical t(1;16) translocations, achieving a successful birth of healthy twins who were homozygous for the translocation [9].

There have been reports stating that t(11;22) has been associated with an increased risk of BC, particularly in premenopausal women [1,14,15]. In their report, Wieland et al. described a family carrying the t(11;22) translocation, in which multiple members had postmenopausal breast cancer. The tumor tissue demonstrated loss of heterozygosity, while the derivative chromosome 22 was retained, suggesting its involvement in oncogenesis [14]. Jobanputra et al. described a family with five BRT carriers with BC, three being premenopausal [15]. While it is advisable for the carriers of this BRT to undergo BC surveillance, inconsistent association of t(11;22) with BC in some studies, suggesting variable penetrance, warrants further investigation into its oncogenic potential [3].

The extraordinary rarity of this case reflects the confluence of rare events: a marriage between two identical translocation carriers, the one in 196 (14x14) probabilities of production of a homozygous offspring, and 10% likelihood of phenotypic normalcy [7,9,10]. The case challenges classical assumptions about homozygous translocations, suggesting compensatory mechanisms, such as redundant gene function, favorable breakpoint positioning, or epigenetic regulation, as proposed by Tapia-Páez et al. [4]. Nonetheless, the absence of whole-genome sequencing or functional assays, along with ideal tests such as Spectral Karyotyping (SKY) and Multicolour Banding (M-BAND) not being performed, represents a limitation of this report. Future studies using these approaches may offer a more comprehensive understanding.

## Conclusions

This case report describes a 31-year-old female with primary infertility, identified as a phenotypically normal homozygous carrier of the most common recurrent non-Robertsonian translocation, an extraordinarily rare. This case emphasizes the importance of IVF and PGT to overcome reproductive challenges. Comprehensive genetic counseling is vital to address reproductive concerns, especially regarding alternative options like donor egg, which may be suggested when IVF with PGT-SR does not result in euploid embryos for transfer, thereby reducing the emotional and financial burden on couples. Moreover, genetic counseling is essential to discuss oncologic and consanguinity-related concerns. Additionally, this observation provides scientific insight into possible mechanisms that may allow for phenotypic normalcy despite homozygosity. Together, these clinical and biological perspectives highlight both the management considerations for affected individuals and the broader implications for understanding complex chromosomal rearrangements.
